# Serum protein profiles predict coronary artery disease in symptomatic patients referred for coronary angiography

**DOI:** 10.1186/1741-7015-10-157

**Published:** 2012-12-05

**Authors:** William A LaFramboise, Rajiv Dhir, Lori A Kelly, Patricia Petrosko, John M Krill-Burger, Christin M Sciulli, Maureen A Lyons-Weiler, Uma R Chandran, Aleksey Lomakin, Robert V Masterson, Oscar C Marroquin, Suresh R Mulukutla, Dennis M McNamara

**Affiliations:** 1 University of Pittsburgh, Department of Pathology, 5230 Centre Avenue, Pittsburgh, PA 15232, USA; 2 University of Pittsburgh, Department of Biomedical Informatics, 5150 Centre Avenue, Pittsburgh, PA 15232, USA; 3 Materials Processing Center, Massachusetts Institute of Technology, Cambridge, MA 02139, USA; 4 Prevencio LLC, 454 N 34th Street, Seattle, WA 98103, USA; 5 University of Pittsburgh, Division of Cardiology, Department of Medicine, 200 Lothrop Street, Pittsburgh, PA 15213, USA; 6 UPMC Heart and Vascular Institute, 200 Lothrop Street, Pittsburgh, PA 15213, USA; 7 University of Pittsburgh, Department of Epidemiology, Graduate School of Public Health, Pittsburgh PA, 15213, USA

**Keywords:** atherosclerosis, biomarkers, cardiac catheterization, coronary angiography, coronary stenosis, multiplex proteomics

## Abstract

**Background:**

More than a million diagnostic cardiac catheterizations are performed annually in the US for evaluation of coronary artery anatomy and the presence of atherosclerosis. Nearly half of these patients have no significant coronary lesions or do not require mechanical or surgical revascularization. Consequently, the ability to rule out clinically significant coronary artery disease (CAD) using low cost, low risk tests of serum biomarkers in even a small percentage of patients with normal coronary arteries could be highly beneficial.

**Methods:**

Serum from 359 symptomatic subjects referred for catheterization was interrogated for proteins involved in atherogenesis, atherosclerosis, and plaque vulnerability. Coronary angiography classified 150 patients without flow-limiting CAD who did not require percutaneous intervention (PCI) while 209 required coronary revascularization (stents, angioplasty, or coronary artery bypass graft surgery). Continuous variables were compared across the two patient groups for each analyte including calculation of false discovery rate (FDR ≤ 1%) and *Q *value (*P *value for statistical significance adjusted to ≤ 0.01).

**Results:**

Significant differences were detected in circulating proteins from patients requiring revascularization including increased apolipoprotein B100 (APO-B100), C-reactive protein (CRP), fibrinogen, vascular cell adhesion molecule 1 (VCAM-1), myeloperoxidase (MPO), resistin, osteopontin, interleukin (IL)-1β, IL-6, IL-10 and N-terminal fragment protein precursor brain natriuretic peptide (NT-pBNP) and decreased apolipoprotein A1 (APO-A1). Biomarker classification signatures comprising up to 5 analytes were identified using a tunable scoring function trained against 239 samples and validated with 120 additional samples. A total of 14 overlapping signatures classified patients without significant coronary disease (38% to 59% specificity) while maintaining 95% sensitivity for patients requiring revascularization. Osteopontin (14 times) and resistin (10 times) were most frequently represented among these diagnostic signatures. The most efficacious protein signature in validation studies comprised osteopontin (OPN), resistin, matrix metalloproteinase 7 (MMP7) and interferon γ (IFNγ) as a four-marker panel while the addition of either CRP or adiponectin (ACRP-30) yielded comparable results in five protein signatures.

**Conclusions:**

Proteins in the serum of CAD patients predominantly reflected (1) a positive acute phase, inflammatory response and (2) alterations in lipid metabolism, transport, peroxidation and accumulation. There were surprisingly few indicators of growth factor activation or extracellular matrix remodeling in the serum of CAD patients except for elevated OPN. These data suggest that many symptomatic patients without significant CAD could be identified by a targeted multiplex serum protein test without cardiac catheterization thereby eliminating exposure to ionizing radiation and decreasing the economic burden of angiographic testing for these patients.

## Background

Coronary heart disease is the most prevalent chronic disease and the leading cause of death in the US, with more than half a million newly diagnosed coronary artery disease (CAD) patients annually [1,2]. Cardiac catheterization and coronary angiography are often necessary for definitive evaluation of coronary artery anatomy, the presence of coronary atherosclerosis and to determine the need for interventional therapy. Despite the high prevalence of CAD, approximately half of patients undergoing invasive cardiac catheterization either have no significant coronary lesions or do not require any mechanical or surgical form of revascularization [3-5]. Thus, the procedure could be eliminated in many cases if alternative, non-invasive tools were available to assess the presence or absence of significant CAD and confirm the need for angiography.

Clinical assessment of CAD represents a significant medical and economic challenge comprising more than a million coronary angiograms annually in the US alone with demographics of aging and obesity forecasting growing demand [2-5]. The risk and expense of cardiac catheterization (ionizing radiation, contrast media, morbidity) and the large number of patients with normal coronary arteries or 'non-significant' CAD undergoing invasive angiography warrant development of alternative tests for CAD without cardiac catheterization [5]. While progress has been made using non-invasive computed tomography (CT) particularly for its negative predictive value, CT incorporates significant exposure to ionizing radiation with considerably lower resolution than catheterization-based angiography [6].

Efforts to identify circulating biomarkers for CAD have shown promise by interrogating transcriptional profiles of patient blood cells and plasma for unique mRNA and microRNA signatures [7,8]. Since extracellular RNA undergoes rapid degradation, prospective mRNA signatures were derived predominantly from nucleated blood cells while the miRNAs identified in plasma were likely protected in circulating vesicles or bound to protective protein complexes [9]. Consequently, the utility of RNA as an indicator of CAD is constrained by its selective cell source in the bloodstream, the friability of the ribonucleotide targets and the arduous process of RNA capture, purification, amplification and analysis. In contrast, circulating proteins are more stable than RNA in blood and serum with several individual markers identified previously as prospective biomarkers for the presence of atherosclerosis, myocardial infarction, heart failure, or markers of pathways involved in these cardiac conditions such as inflammation, thrombosis, plaque stability, and oxidative stress, for example, troponin C, pro-brain natriuretic peptide (BNP) and C-reactive protein (CRP) [10,11]. However, circulating biomarkers have proven to be of limited value in clinical tests to diagnose coronary artery disease antecedent to a cardiac event, primarily because most studies have focused on single or at most a few markers to make the diagnosis [12]. The difficulty in identifying predictive factors of CAD in blood or serum is compounded by the multifactorial etiology of coronary artery disease which makes early diagnosis by a single, endpoint marker unlikely prior to activation of a common ischemic pathway or until significant myocardial compromise has occurred.

The hypothesis underlying the current study was that coronary artery disease status can be assessed via individual and/or combinatorial protein changes in serum that assess multiple pathways of atherosclerosis as a low-risk, non-invasive approach for screening of symptomatic patients, that is, patients referred for cardiac catheterization. The study targeted patients who were referred for a clinically-indicated cardiac catheterization from either the emergency room or outpatient cardiac clinic in a major metropolitan center who presented with symptoms of heart disease. All patients had blood drawn prior to coronary angiography and revascularization. By analyzing a compendium of 41 circulating protein targets associated with atherogenesis, inflammation, thrombosis, and plaque vulnerability, we discovered 12 diverse proteins expressed across a broad dynamic range that were significantly different concomitant with the need for these patients to undergo therapeutic intervention including stent placement, angioplasty, or coronary artery bypass graft surgery (CABG). We also tested multiplex biomarker signatures for the potential to discriminate patients lacking significant coronary artery disease from patients with CAD requiring corrective interventional therapy. In particular, the ability to rapidly and decisively rule out clinically significant coronary artery disease using a potentially low cost, low risk blood test in even a small percentage of patients with normal coronary arteries could be highly beneficial.

## Methods

### Patient group

Samples comprised serum from among 359 subjects referred for a clinically-indicated cardiac catheterization for symptoms of CAD. The study was performed according to the Department of Health and Human Services Code of Federal Regulations (45 CFR 46) for the protection of human research subjects including ethical considerations consistent with the Office for Human Research Protections. Blood was collected following a genetic banking protocol (#990835) approved by the University of Pittsburgh Institutional Review Board (IRB). Only patients who signed the approved IRB voluntary informed consent document for this study were included (11 January 2000 to 21 July 2004). Venous blood (5 ml) was drawn into a red top vacutainer serum tube (Becton Dickinson #366430, Franklin Lakes, NJ, USA) and placed upright 30 to 60 minutes until clot formation. The tubes were centrifuged in a swinging bucket rotor (1,300 g × 20 min) and the serum was pipetted into 1.5 ml cryovials for storage at -80°C. All 359 patients underwent diagnostic coronary angiography and 209 required interventional therapy comprising stent placement, angioplasty or CABG. The other patients had normal or clinically insignificant coronary artery disease, that is, angiography revealed absence of any vessel obstruction or non-critical, < 50% obstruction in any epicardial vessel. Thus, serum samples comprised one patient group based on clinical symptoms but two outcome groups based on therapeutic intervention.

### Proteomics analysis

Samples underwent a first thaw on ice to apportion into 200 μl aliquots for -80°C storage until analysis. An exploratory study of 56 samples was performed using fluorokine multianalyte profiling (xMAP) of 33 proteins (Luminex 100; Luminex, Austin, TX, USA) to determine serum dilution factors and rule out targets lacking statistical discrimination. The assay used polystyrene microspheres incorporating differing ratios of two fluorophores yielding different spectrally addressed bead sets each conjugated with a biotinylated protein-specific capture antibody. Assays were processed in duplicate in a randomized, blinded manner regarding patient outcomes, including generation of a standard curve using recombinant target proteins. Each 96 well microplate was incubated overnight at 4°C on a microtiter shaker. Wells were washed with buffer (3 ×) and a secondary antibody added to each well for incubation (2 h, room temperature) followed by streptavidin-phycoerythrin (0.5 h, room temperature, agitation). The wells were then washed (2 ×), assay buffer was added, and samples were analyzed using the Bio-Plex suspension array system and Bio-Plex Manager software 4.0 (Bio-Rad Laboratories, Hercules, CA, USA). Absolute quantities were determined by comparison to the five-point standard curve for each analyte.

The Searchlight Protein Array System (Aushon Biosystems, Inc, Billerica, MA, USA) was used to interrogate patient serum samples at 2 different stages (stage 1: 239 samples, 24 analytes; stage 2: 120 samples, 10 analytes). First, 239 samples were evaluated for 24 analytes over concentration ranges defined by the preliminary study of 56 samples. The assay comprised a multiplex sandwich ELISA of monoclonal capture antibodies spotted in planar arrays in 96-well microtiter plates. After serum incubation and washing, a second biotinylated monoclonal antibody to a different site from the capture epitope was introduced and streptavidin-horseradish peroxidase (HRP) subsequently bound to the biotin site. Luminol enhancer/peroxidase solution was added and the HRP catalyzed oxidation of luminol to 3-aminophthalate resulting in light emission at 428 nm. A chemiluminescent image was acquired and processed using a four-parameter curve fit program (SearchLight Array Analyst Software) to compare the experimental samples to the recombinant calibration curve run in parallel wells to derive absolute concentrations adjusted for dilution and quality values.

The largest SearchLight panel simultaneously evaluated seven analytes diluted 1:1 (volume/volume) (dilution factor (df) = 2 ×) in assay buffer (RPMI1640 without phenol red + 10% heat inactivated FBS) including interferon γ (IFNγ), interleukin 1β (IL-1β), IL-6, IL-10, matrix metalloproteinase protein 1 (MMP1), thrombomodulin (TM) and tumor necrosis factor α (TNFα). Leptin, platelet endothelial cell adhesion molecule 1 (PECAM-1), endothelial leukocyte adhesion molecule 1 (E-selectin), monocyte chemoattractant protein 1 (MCP-1), MMP7 and vascular cell adhesion molecule 1 (VCAM-1) were assayed together at 25 × dilution factor. Tissue inhibitor of metalloproteinase 1 (TIMP-1), fibrinogen, resistin, leukocyte selectin (L-selectin) and myeloperoxidase (MPO) (df = 1,000 ×) were analyzed in a five-analyte panel. Adiponectin (ACRP-30) and C-reactive protein (CRP) were assayed together at a dilution factor of 5,000 ×. Apolipoprotein A1 (APO-A1, df = 50,000 ×), apolipoprotein B100 (APO-B100, df = 10,000 ×), osteopontin (OPN, df = 10 ×) and N-terminal fragment protein precursor brain natriuretic peptide (NT-pBNP, df = 2 ×) were interrogated independently.

A second stage study of 120 additional serum samples was repeated twice to validate the previous findings from the 239 sample set and to test assay reproducibility across different reagent and planar array lots. The sample prep, quality control (QC), methodological protocols for recombinant protein calibration profiles, serial dilutions and serum assays were performed as before but using fewer panels and smaller analyte configurations. These included MPO, fibrinogen and resistin (df = 1,000 ×) in a three-multiplex configuration, ACRP-30 and APO-B100 together (df = 10,000 ×), MMP7 and VCAM-1 together (df = 25 ×) and osteopontin (df = 10 ×), IFN-γ (df = 2 ×) and IL-1β (df = 2 ×) separately.

### Statistical analysis

Patients were operationally defined as 'symptomatic' by referral for a clinically indicated catheterization. Based on coronary angiography outcome, the serum samples were classified from patients with 'normal' coronary arteries, that is, no clinically significant coronary artery disease (n = 150) or patients with coronary disease requiring therapy including stent placement, angioplasty or CABG (n = 209). The hypothesis undergoing statistical testing was that serum proteins were significantly different between the two patient outcome classifications. Statistical analysis was initially performed on 239 samples in stage 1 evaluated for 24 analytes comprising 101 serum samples from patients with clinically normal coronary arteries and 138 samples from patients requiring percutaneous intervention (PCI). These samples were also used to develop and train a scoring function algorithm. A second stage validation study (n = 120) interrogating 10 analytes was subsequently performed to validate the algorithm. Results from all of these studies were combined for statistical comparison. Continuous variables were compared (Partek Genomics Suite, St. Louis, MO, USA) using the unpaired Student's t test across the two patient groups for each analyte including calculation of a false discovery rate (FDR) and *Q *value as the minimum positive FDR for rejecting a statistic [13]. Significant differential expression of proteins was defined at an FDR of ≤ 1% with *Q *= 0.01 and statistical significance for *P *values was adjusted to ≤ 0.01. Categorical variables were compared using the Pearson's χ^2 ^test.

### Algorithm development and validation of selected markers

Data for all 24 markers interrogated in stage 1 were evaluated as randomized, multimarker signatures to classify patients with CAD requiring treatment versus patients without clinically significant CAD. A scoring function (SF) algorithm was generated for all protein combinations as disease 'signatures' including 24 'artificial' markers derived by randomly scrambling the data (see Additional File 1). The SF for each signature was a linear combination of natural logarithms of marker concentrations generated by iterative computation. Monte Carlo optimization determined coefficients that provided highest diagnostic accuracy, that is, specificity (SP: identification of negatives for significant CAD) for detecting patients with normal coronary arteries while maintaining 95% sensitivity (SN: identification of CAD requiring interventional therapy) for patients with coronary artery disease. We ranked > 2 million combinations of 2 to 5 marker signatures comprising the 24 actual and 24 'artificial' markers for ability to classify patients since combinations of 6 or more proteins with high classification strength commonly contained an artificial marker (see Additional file 2 Table S1). For each signature of 2 to 5 markers, the top 50 panels with highest SP for normal (while correctly detecting at least 95% of the CAD patients) underwent cross validation testing where 80% of participants were randomly selected as a training set to build the optimal SF and the remaining 20% of participants were thenclassified using this SF. The crossvalidation procedure was repeated 500 times and average SP and SN were used to identify best performing signatures.

Independent verification of the scoring function algorithm was performed in 2 repeated studies of 120 serum specimens from an additional cohort of symptomatic patients with clinical characteristics matching the previous 239 patients. Concentration values for these samples were entered into the algorithm in a macro subroutine program using the offset, coefficients and cut-offs to detect CAD based on patient outcome. The results of the 120-sample validation study were compared to the diagnostic classification of each patient after coronary catheterization and follow-up therapy to determine the sensitivity and specificity of prospective signatures.

## Results

Diagnostic coronary angiography revealed that 209 of the patients in this study exhibited significant coronary artery disease requiring therapeutic intervention while 150 patients did not exhibit clinically significant coronary artery disease despite symptoms or other findings that led to referral for cardiac catheterization. These two distinct outcome groups were otherwise identical upon admission regarding clinical symptoms and physical characteristics including gender, diabetic status, smoking history, body surface area, basal metabolic rates, cholesterol, LDL and creatinine values (see Table 1). Among continuous variables, there were small albeit significant differences in age, HDL levels and ejection fraction between groups; but the differences were of minimal diagnostic value and all patients required coronary angiography. Regarding categorical variables, there were no significant differences in gender or diabetes between the two groups; however, the number of patients with hypertension was significantly higher in the CAD group.

**Table 1:** Clinical characteristics of the patient groups

Characteristic	Average NOR	SD	N	Average CAD	SD	N	*P *value
HDL	50.5	18.2	85	41.6	9.9	141	< 0.001
Age	57.9	10.5	149	62.8	10.6	203	< 0.001
EF%	57.8	8.4	133	53.1	10.9	165	< 0.001
BMI	30.6	7.6	149	29.8	5.1	204	NS
BSA	2.1	0.3	149	2.0	0.2	204	NS
CHOL	196.7	36.2	87	192.2	45.9	140	NS
CREAT	1.0	0.7	100	1.0	1.0	123	NS
HT	171.4	9.5	148	171.6	10.1	204	NS
LDL	120.9	37.8	82	120.5	40.5	132	NS
WT	90.6	21.9	148	87.5	15.6	204	NS
	**NOR**			**CAD**			
HYPTX	76 Y	74 N	150	125 Y	79 N	204	< 0.01
Gender	81 M	69 F	150	126 M	78 F	204	NS
DIAB	16 Y	134 N	150	31 Y	173 N	204	NS

Average and SD (standard deviations) for NOR patients (normal patients without clinically significant coronary artery disease) and CAD patients (patients with coronary artery disease requiring therapeutic percutaneous intervention) are shown. N = number of samples for which parameters were available. P = statistical *P *values for individual continuous parameters between the NOR and CAD groups with adjusted false discovery rate (FDR) cut-off for significance ≤ 0.01. *P *values for categorical values were determined by Pearson's χ^2 ^test. NS indicates that no significant statistical differences were detected. M/F indicates the number of males and females in each group with age expressed in years, WT (weight) in kg and HT (height) in cm.

DIAB (diabetes) and HYPTX (hypertension) reported as the number of positive (Y) or negative (N) patients per group.

BSA = body surface area calculated in m^2^; BMI = body mass index (weight in kg/height in m^2^); CREAT = creatinine, mg/dl; CHOL = cholesterol, mg/dl; EF% = ejection fraction, percentage; HDL = high-density lipoprotein, mg/dl; LDL = low-density lipoprotein, mg/dl.

**Figure 1:**
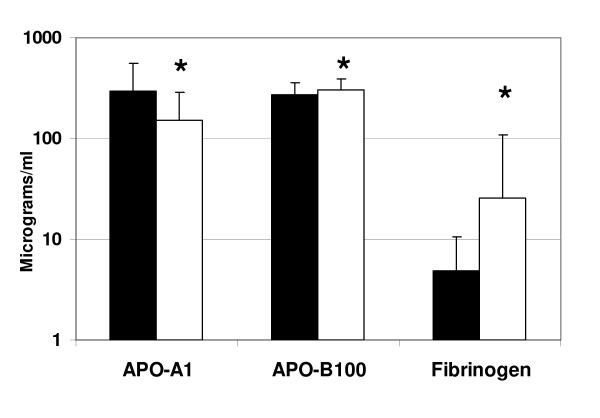
**Significant differences in apolipoprotein A1 (APO-A1), apolioprotein B100 (APO-B100) and fibrinogen in serum from normal and coronary artery disease (CAD) patients**. Solid bars are values expressed as average plus 1 SD for APO-A1, APO-B100, and fibrinogen obtained from patients without clinically significant coronary artery disease (normal, n = 150) based on coronary angiographic evaluation. Open bars are results obtained from patients with CAD requiring interventional therapy (n = 209). Values are expressed in μg/ml on a logarithmic ordinate scale and each was significantly different (*) between groups (see Table 2 for individual *P *values).

**Figure 2:**
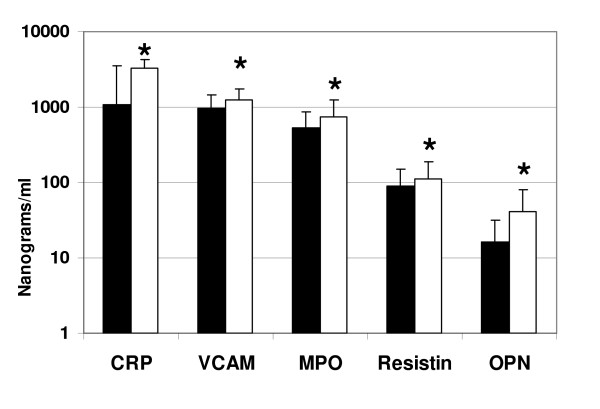
**Significant differences in vascular cell adhesion molecule, myeloperoxidase, C-reactive protein, resistin and osteopontin in serum from normal and coronary artery disease (CAD) patients**. Normal and CAD data are displayed according to Figure 1 but expressed in ng/ml on a logarithmic ordinate scale. All comparisons represent significant statistical differences delineated in Table 2 (*) for vascular cell adhesion molecule (VCAM-1), myeloperoxidase (MPO), C-reactive protein (CRP), resistin and osteopontin (OPN).

**Figure 3:**
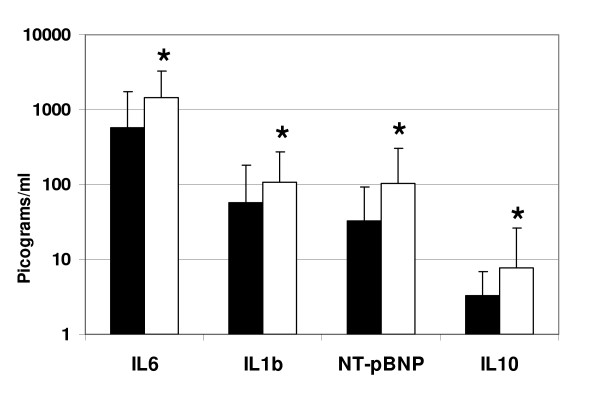
**Significant differences in interleukin (IL)-6, IL-1β, IL-10 and N-terminal fragment pro-brain natriuretic peptide (NT-pBNP) in serum from normal and coronary artery disease (CAD) patients**. Normal and CAD data are displayed according to Figure 1 but expressed in pg/ml on a logarithmic ordinate scale. All comparisons represent significant statistical differences (*) reported in Table 2 for IL-6, IL-1β, IL-10 and NT-pBNP.

All serum samples were collected, processed, stored and analyzed in an identical manner to limit the effect of preanalytic variability including differential protein degradation among specimens. Significant differences were detected in 12 serum proteins (*Q *value = 0.01; *P *< 0.01) between patients diagnosed as having CAD requiring intervention and those with non-significant CAD after diagnostic coronary angiography. The differences detected in the stage 1 study (n = 239) were reinforced by the additional samples from the validation study (n = 120) (see Table 2). APO-A1 and APO-B100 were among the highest expressed proteins overall averaging approximately 300 μg/ml of serum (Figure 1). APO-A1 fell significantly in patients with significant CAD versus non-significant CAD while APO-B100 was significantly increased. Within the same concentration range, fibrinogen was present at levels typically exceeding 1 μg/ml with values fivefold higher in patients with significant CAD (Figure 1). At serum concentrations from 10 ng to 1 μg/ml serum, five proteins were significantly higher in CAD patients. Specifically, CRP, VCAM-1, MPO, resistin and osteopontin were 1.2 to 3.1-fold higher than patients with non-significant CAD (Figure 2). Four analytes, IL-6, IL-1β, IL-10 and NT-pBNP were significantly higher in the CAD group among analytes detected in a range from 1 pg/ml to 1 ng/ml (Figure 3). There were no significant statistical correlations between any of these 12 analytes and age, ejection fraction or hypertension status which were significantly but incrementally different between the patient outcome groups. No other analytes among the 41 interrogated targets were significantly altered between the 2 groups of patient samples using either bead-based or planar platforms.

**Table 2:** Multiplex proteomics analysis of the coronary artery disease (CAD) and normal (NOR) patient groups

	Average NOR	SD	N	Average CAD	SD	N	*P *value
OPN	16.4 ng	15.3 ng	149	40.6 ng	38.1 ng	206	< 0.001
VCAM	980.7 ng	493.8 ng	149	1,266.5 ng	492.5 ng	206	< 0.001
APO-A1	293.7 μg	257.4 μg	91	152.5 μg	134.5 μg	135	< 0.001
IL-6	0.6 ng	1.1 ng	101	1.5 ng	1.8 pg	135	< 0.001
MPO	538.2 ng	333.9 ng	147	731.1 ng	532.5 ng	206	< 0.001
IL-1β	56.9 pg	124.0 pg	149	109.0 pg	164.0 pg	206	< 0.005
NT-pBNP	33.3 pg	59.4 pg	91	103.2 pg	204.5 pg	135	< 0.005
Fibrinogen	4.8 μg	5.8 μg	149	25.6 μg	81.6 μg	206	< 0.005
APO-B100	273.2 μg	80.6 μg	149	300.6 μg	93.0 μg	206	< 0.005
Resistin	89.5 ng	63.0 ng	149	110.7 ng	77.0 ng	206	< 0.005
CRP	1.1 μg	2.4 μg	149	3.3 μg	9.7 μg	201	< 0.005
IL-10	3.3 pg	3.7 pg	91	7.6 pg	18.4 pg	135	< 0.005
MMP1	4.7 ng	2.3 ng	91	5.3 ng	2.4 ng	135	0.015 (NS)
Leptin	13.6 ng	18.0 ng	100	10.0 ng	16.0 ng	133	0.027 (NS)
ACRP30	5.9 μg	3.9 μg	149	5.2 μg	4.0 μg	206	0.027 (NS)
MMP7	4.8 ng	3.3 ng	149	5.2 ng	2.4 ng	206	0.045 (NS)
TNFα	22.7 pg	77.1 pg	91	14.7 pg	19.3 pg	135	0.059 (NS)
IFNγ	3.6 pg	10.8 pg	149	4.7 pg	10.0 pg	206	0.069 (NS)
L-selectin	1.1 μg	0.2 μg	91	1.1 μg	0.3 μg	135	0.074 (NS)
MCP1	3.1 ng	3.7 ng	91	3.4 ng	3.4 ng	135	0.114 (NS)
PECAM-1	44.0 ng	32.1 ng	57	45.8 ng	27.2 ng	79	0.116 (NS)
TIMP1	321.3 ng	101.1 ng	91	327.2 ng	86.1 ng	135	0.116 (NS)
E-selectin	33.2 ng	19.2 ng	91	34.1 ng	16.6 ng	135	0.123 (NS)
TM	1.4 ng	0.8 ng	91	1.4 ng	0.4 ng	135	0.128 (NS)

Protein concentrations are reported in pg, ng or μg per ml of serum with adjusted *P *values for false discovery rate (FDR) at a cut-off for statistical significance between the NOR and CAD groups = 0.01. NS indicates that no significant statistical differences were detected with the calculated *P *values provided in parenthesis.

ACRP-30 = adiponectin; APO-A1 = apolipoprotein A1; APO-B100 = apolipoprotein B100; CRP = C-reactive protein; E-selectin = endothelial leukocyte adhesion molecule 1; IFNγ = interferon γ; IL = interleukin; L-selectin = leukocyte selectin; MCP-1 = monocyte chemoattractant protein 1; MMP matrix metalloproteinase; MPO = myeloperoxidase; NT-pBNP = N-terminal fragment protein precursor brain natriuretic peptide; OPN = osteopontin; PECAM-1 = platelet endothelial cell adhesion molecule 1; TIMP-1 = tissue inhibitor of metalloproteinase 1; TM = thrombomodulin; TNFα = tumor necrosis factor α; VCAM-1 = vascular cell adhesion molecule 1.

**Figure 4:**
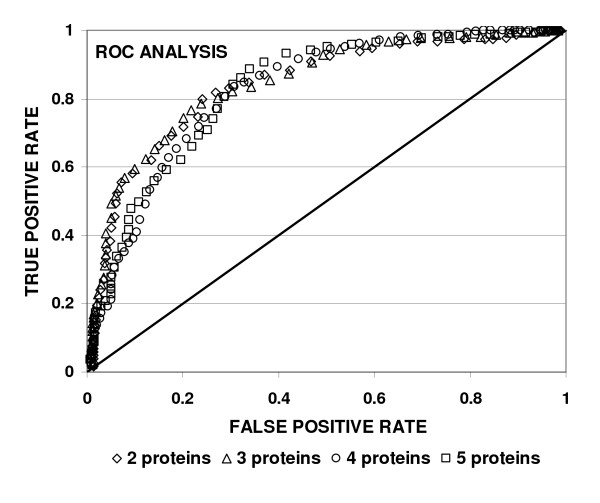
**Receiver operating characteristics (ROC) for 2 to 5 protein panels for identification of normal patients with 95% specificity for detection of coronary artery disease (CAD) patients**. The ROC curves are derived from 4 separate panels optimized to detect 101 normal patients (true positives in this figure) at highest specificity while maintaining a sensitivity of 95% for patients with CAD (138 samples). The ROC curves are obtained by iteratively testing each biomarker panel for classification of a randomly excluded portion (20%) of the dataset. The areas under the curve (AUC) were comparable as indicated in the curves for two proteins (osteopontin (OPN) and resistin: AUC = 0.839), three proteins (OPN, resistin, apolioprotein B100 (APO-B100): AUC = 0.845), four proteins (OPN, resistin, matrix metalloproteinase 7 (MMP7) and interferon γ (IFNγ): AUC = 0.839) and five proteins (OPN, resistin, MMP7, IFNγ and C-reactive protein (CRP): AUC = 0.827). The predicted specificity for detection of normal patients at 95% sensitivity for CAD patients was two proteins = 50%, three proteins = 52%, four proteins = 63% and five proteins = 64%.

We identified 14 multiplex signatures of 2 to 5 proteins each with the highest acuity to detect patients without significant CAD (22.6% to 58.4% SP) while detecting 95% of the significant CAD group (95% SN) in the stage 1 study (see Additional file 2 Table S1). A total of 11 distinct proteins were shared among the 14 signatures with osteopontin (14 of 14), and resistin (10 of 14) most frequently represented. There was a trend for protein signatures with increased numbers of analytes to detect more normal patients at a fixed sensitivity for CAD patients (95%) (two proteins = 39.3% ± 0.3% vs five proteins = 50.0% ± 0.01% of normal patients). However, a performance plateau was reached at five biomarkers based on crossvalidated classifier performance and the frequency of appearance of 'artificial' markers in test signatures exceeding five proteins. Receiver operating characteristics analysis indicated that these signatures were effective in discerning patients without significant CAD. The area under the curve (AUC) for the top signatures ranged from a low of 0.839 ± 0.028 (mean ± SD) for a two-protein signature (OPN, resistin) to a maximum AUC of 0.845 using three biomarkers (OPN, resistin, APO-B100) (Figure 4). These ROC curves were compared to those generated by the Bayesian compound covariate predictor algorithm for the same data set. The area under the curve using the scoring function algorithm exceeded that obtained by the Bayesian predictor in every case. A clinical validation test of 120 additional serum samples (49 normal, 71 patients requiring intervention) was performed to test the performance of the scoring function algorithm. In two separate studies, the best performing multiplex signatures contained five proteins (OPN, resistin, MMP7, IFNγ with either CRP or ACRP-30) and were able to correctly classify 88% and 92% of patients requiring percutaneous intervention while delineating 33% and 36% of the patients with normal coronary arteriograms.

## Discussion

Proteins were selected for evaluation in this study based on their roles in mechanisms underlying atherogenesis, atherosclerosis and plaque instability including vascular inflammation, thrombosis, aberrant lipid regulation, metabolism hormones, and vascular smooth muscle and extracellular matrix (ECM) remodeling [14]. The 41 preliminary targets we interrogated were restricted by availability of monoclonal antibody pairs optimized for use in the commercial assay platforms. IL-1β, IL-6, IL-10 and VCAM-1, were significantly elevated in patients with CAD in the present study consistent with an injury-induced, inflammatory response [15,16]. Elevated IL-1β and IL-6 have been associated previously with acute phase protein induction and may explain the concomitant significant increases in fibrinogen and CRP concentration we detected. CRP has long been proposed as a surrogate marker for inflammatory mediators in predicting coronary events while NT-pBNP has been used as an indicator of left ventricular dysfunction in CAD patient cohorts comparable to this study [11,17,18]. Both analytes were significantly elevated in the present study among patients requiring therapeutic intervention and CRP was among the best single molecule classifiers delineating 19% of normal samples while detecting 95% of the patients with significant CAD.

Significant reciprocal changes were detected in APO-A1 and APO-B100 in CAD patients consistent with reports defining aberrant lipid transport and accumulation as contributory to atherosclerosis [19]. Mutations in the APO-B100 gene cause autosomal dominant, hereditary familial hypercholesterolemia and premature coronary artery disease due to defective ligand binding [19,20]. At the same time, elevated APO-A1 is associated with a cardioprotective effect and enhancement of APO-A1 expression has been proposed as a therapeutic strategy to inhibit atheroma formation [19,21]. The increased APO-B100 and decreased APO-A1 levels in our patients requiring PCI versus normal controls support these previous findings. Myeloperoxidase was also significantly increased in CAD patients associated with its role as a catalyst for lipid peroxidation at inflammation sites and as a marker of plaque instability [22,23]. Resistin levels were elevated in the PCI patients indicative of 1) metabolic shifts in lipid utilization and adipogenesis and/or 2) an inflammatory response with resistin secreted from macrophages concomitant with the release of proinflammatory cytokines [24].

Many targets traditionally associated with vascular smooth muscle and ECM remodeling were not significantly altered among these patient groups including matrix metalloproteinases 1, 2, 3, 7, 9 and tissue inhibitors of metalloproteinases 1, 2, 3 and 4. Only osteopontin, which acts as a negative regulator of calcification in bone remodeling, was elevated within this category with the rejoinder that OPN also may act as a chemokine in the cell mediated type 1 immune response associated with inflammatory cell accumulation rather than as a substrate for cell adhesion [25]. Thus, the proteins that demarcated our patient outcome groups were predominantly associated with processes of inflammation and lipid regulation rather than cellular aggregation and ECM remodeling. However, we recognize that the domain of proteins susceptible to interrogation in this study was limited to analytes for which high affinity antibody pairs precisely characterized to two different epitopes were available. The involvement of additional proteins and pathways associated with CAD will likely be reinforced and/or revealed as the inventory of immunoassays becomes more comprehensive.

Our data indicate multiplex proteomics analyses using monoclonal antibodies provide relevant information regarding circulating serum analyte concentrations when assayed at a dilution that allows direct comparison to parallel recombinant calibration standards. Advantages include small serum volumes (< 100 μl) collected by standard clinical protocols, rapid turnaround times (minutes to hours), high sensitivity (pg) and a broad dynamic range (8 logs). Disadvantages include high assay cost, limited target availability and poor concurrence of concentration measurements across dilutions and commercial platforms associated with variations in antibodies, buffers, diluents and capture structures. In the present study, 15 targets were tested at identical serum dilutions using bead-based (Luminex) and planar (Aushon) technologies in 56 identical samples, albeit with different aliquots and in serial studies. A total of 12 assays concurred in detection of statistically significant differences between the 2 patient outcome groups. These results suggest that multiplex immunochemical assays of serum may provide information of diagnostic relevance but that protocols and reagents must be optimized and standardized prior to routine clinical application.

The results of this study were somewhat surprising both for discovery of unique proteins as discriminants of CAD and for the absence of statistically significant differences in many targets with established roles in atherosclerosis. For example, osteopontin has been only indirectly associated with atherosclerosis yet exhibited the greatest statistical difference between patient groups (*P *= 1.75 × 10^-12^). Osteopontin was first identified as a sialoprotein from mineralized bone matrix and only recently was associated with calcification of plaques in cardiac valves and vessels [25-27]. Similarly, resistin has been linked only indirectly to CAD through a role in metabolic homeostasis and insulin sensitivity [28]. On the other hand, multiple growth factors (VEGF, leptin, ghrelin), lipoproteins (APO-A2, E, serum amyloid A: SAA), cell adhesion molecules (thrombospondin, PECAM-1, ICAM-1, selectins E, L, P) and MMP and TIMP targets associated with ECM remodeling exhibited no statistically significant differences. There are several potential explanations for the latter findings: (1) a rigorous statistical standard was utilized to avoid multiple testing errors and while MMP1, MMP7, ACRP-30, and leptin were borderline for statistical significance (*P *= 0.015, 0.045, 0.027, 0.027 respectively) they failed to reach the *Q *= 0.01 level established for significance with adjusted *P *values ≤ 0.01 in this study; (2) serum may not be an effective transducer of deleterious protein changes participating in structural rearrangements within the coronary vascular anatomy and the extracellular matrix; and (3) the patients comprised a diverse range of coronary obstruction and plaque vulnerability since they were selected for symptoms upon emergent presentation requiring diagnostic coronary angiography without the occurrence of a clinically obvious myocardial infarct or an 'event'. A subset of patients selected for advanced disease might reveal additional protein changes but stray from the intended focus of this study.

A scoring function algorithm was developed, tested and validated for the ability to classify patients symptomatic for heart disease consistent with the outcome of coronary angiography studies and need for interventional therapy. We minimized selection bias by testing a hypothesis driven biomarker panel and avoided overfitting by performing cross validation and follow-up testing utilizing additional serum samples from the cohort. The algorithm was designed to be 'tuned' to increase the sensitivity for capture of patients who required coronary revascularization at the expense of detecting fewer patients who did not require coronary revascularization. All serum signatures with highest classification strength from the training trial (239 samples) included osteopontin and signatures containing 4 or 5 proteins performed best during both training and validation phases. The most efficacious protein signature in validation studies comprised OPN, resistin, MMP7 and IFNγ as a four-marker panel while the addition of either CRP or ACRP-30 yielded comparable results in five protein signatures.

Further validation of the diagnostic accuracy of this approach will require extensive testing in greater numbers of patients at multiple locations as well as a prognostic cohort. It is possible that inclusion of clinical variables and risk factors in the biomarker algorithm or using the algorithm as part of a clinical scoring system will enhance both the fidelity and the efficacy of this approach for diagnostic purposes [29,30]. In that context, we calculated 10-year Framingham Coronary Heart Disease (CHD) Risk Scores for patients where all clinical variables (gender, age, total cholesterol, HDL, systolic blood pressure, smoking and diabetes status, use of antihypertensive medication) were acquired prior to coronary angiography [31]. This represented 91 patients who subsequently required therapeutic revascularization (CAD: CHD Score = 14.9 ± 8.5) versus 63 patients who were determined to be free of significant coronary artery disease (no CAD: CHD score = 10.2 ± 6.7). The Framingham CHD scores were statistically different between groups (*P *< 0.001, unpaired Student's t test) but they classified only 16% of the subjects without significant CAD (10 of 63) at a 95% sensitivity for patients with CAD. In contrast, our algorithm incorporating serum values for OPN, RES, CRP, MMP7 and IFNγ identified 63% of the subjects without significant CAD (40 of 63) at 95% sensitivity for patients with CAD. Thus, our multiplex serum protein classifier correctly identified four times as many patients as the Framingham index. The strength of adding clinical variables to our scoring function remains to be determined, but the ability to exempt significant numbers of patients with normal coronary arteries or non-significant CAD from cardiac catheterization with a blood test represents a major economic and health benefit given the growing epidemic of CAD in the US and abroad.

## Conclusions

The results of the present study indicate that a serum, multiplex biomarker assay may provide a clinically useful tool in combination with other standardized clinical tests to facilitate the decision-making process for performing cardiac catheterization in symptomatic patients. The development of highly sensitive monoclonal antibodies to additional pertinent targets along with the formulation of novel predictive algorithms will likely improve the efficacy of this approach. The long-term potential benefits include reduced patient exposure to ionizing radiation and minimization of the rapidly escalating healthcare costs associated with the use of invasive angiography to rule out coronary artery disease.

## Abbreviations

ACRP-30: adiponectin; APO: apolipoprotein; AUC: area under the curve; CABG: coronary artery bypass graft surgery; CAD: coronary artery disease; CHD: coronary heart disease; CRP: C-reactive protein; CT: computed tomography; ECM: extracellular matrix; E-selectin: endothelial leukocyte adhesion molecule; ELISA: enzyme linked immunosorbant assay; FDR: false discovery rate; HDL: high-density lipoprotein; HRP: horseradish peroxidase; IFN: interferon; IL: interleukin; IRB: institutional review board; LDL: low-density lipoprotein; L-selectin: leukocyte selectin; MCP: monocyte chemoattractant protein; MMP: matrix metalloproteinase protein; PCI: percutaneous intervention; QC: quality control; ROC: receiver operator characteristics; SF: scoring function; SN: sensitivity; SP: specificity; MPO: myeloperoxidase; NT-pBNP: N-terminal fragment protein precursor brain natriuretic peptide; OPN: osteopontin; PECAM: platelet endothelial cell adhesion molecule; RES: resistin; SAA: serum amyloid A; TIMP: tissue inhibitor of metalloproteinase; TM: thrombomodulin; TNF: tumor necrosis factor; VCAM: vascular cell adhesion molecule.

## Competing interests

The University of Pittsburgh has applied for a patent related to the content of this manuscript including DMMcN, OCM, RVM, SRM, AL and WALaF as co-inventors. WALaF and RVM are founders and stockholders in Prevencio LLC, which provided partial support for performance of the proteomics assays. Neither WALaF nor RVM were directly involved in the selection of samples for analysis, performance of the primary proteomics assays or generation of the raw data reported in this study. RD, LAK, PP, JMK-B, CMS, MAL-W and URC have no competing interests associated with this study.

## Authors' contributions

RD, WALaF, OCM, RVM, SRM and DMMcN conceived the study, developed the experimental design and analyte target list, analyzed the data and wrote the manuscript. OCM, SRM and DMMcN were responsible for acquisition and selection of all serum samples utilized in this study. LAK, JMK-B, MAL-W, CSM and PP performed all laboratory assays under the direction of RD, compiled the database and performed primary statistical analysis. JMK-B, AL, WALaF and URC performed secondary statistical analysis. AL and WALaF developed and tested the scoring function algorithm. All authors have read and approved the final version of this manuscript.

## Pre-publication history

The pre-publication history for this paper can be accessed here:

http://www.biomedcentral.com/1741-7015/10/157/prepub

## Supplementary Material

Additional file 1 **Algorithm development**. The analytical form of the scoring function is provided. The testing that was performed to derive and optimize the algorithm is described including the use of artificial markers and cross validation testing. Click here for file

Additional file 2 **Predictive panel selection**. The process of testing and final derivation of the panels comprising two, three, four or five markers that best predicted the classification outcome of the patients based on coronary angiography is provided. Click here for file
