# Supplementary material for: Serum protein profiles predict coronary artery disease in symptomatic patients referred for coronary angiography
Source: BMC Med. 2012 Dec 5;10:157. doi: 10.1186/1741-7015-10-157 (PMC3566965; doi:10.1186/1741-7015-10-157)
Supplement: Additional file 1 — Algorithm development. The analytical form of the scoring function is provided. The testing that was performed to derive and optimize the algorithm is described including the use of artificial markers and cross validation testing. [file 1741-7015-10-157-S1.DOCX]

**Additional File 1: Algorithm Development**

Each panel of prospective markers was evaluated for its specificity in discriminating serum samples derived from patients without coronary artery disease while maintaining 95% sensitivity in delineating samples from patients with coronary artery disease. An optimal scoring function was developed to obtain the highest detection rate for the patients without clinically significant coronary artery disease.

The analytical form of the scoring function was:

#

where is the concentration of the biomarker in the panel for participant and *A* and *C* are numerical coefficients. Coefficient *C* was included to compensate for the effect of errors when a marker value was relatively small. Coefficient C*_i_* was selected to be 1/10th of the average value of the corresponding biomarker. When score calculated for a participant is positive, that participant is predicted to have CAD and when it is negative, that participant is predicted to be absent CAD. Optimization of the scoring function was performed to detect patients that did not have CAD. This involved Monte Carlo optimization using a Metropolis algorithm, to determine coefficients A*_i_* where coefficient A*_0_* is selected to maintain 95% specificity for classification of patients with CAD. In order to estimate the performance of the scoring function, cross validation testing was performed repeatedly using 80% of the data as the training data set and the remaining 20% of the data was used to test the specificity and sensitivity of the adjusted scoring function. All possible panels for the 24 markers including 24 “artificial” markers obtained by randomly scrambling the data were tested for their ability to discriminate between the two patient groups while successfully identifying 95% of the patients with coronary artery disease requiring percutaneous intervention.
