# Supplementary material for: Serum protein profiles predict coronary artery disease in symptomatic patients referred for coronary angiography
Source: BMC Med. 2012 Dec 5;10:157. doi: 10.1186/1741-7015-10-157 (PMC3566965; doi:10.1186/1741-7015-10-157)
Supplement: Additional file 2 — Predictive panel selection. The process of testing and final derivation of the panels comprising two, three, four or five markers that best predicted the classification outcome of the patients based on coronary angiography is provided. [file 1741-7015-10-157-S2.DOCX]

**Additional File 2: Panel Selection**

Artificial marker values (24 sets with 239 markers/set) were generated by randomly scrambling the data. These were added into the analysis to determine the effectiveness of the scoring function in delineating the best marker panels. The presence of an artificial marker in a top ranked panel indicated that the panel's performance was likely coincidental, under the assumption that an artificial marker cannot predict the likelihood of the target disease. After ranking the performance of the panels via cross-validation analysis, the best performing panels without artificial markers were identified. The top 4 panels that did not include any artificial markers were identified for the 2, 3, and 4 marker panels, and the top 2 panels that did not include any artificial markers were identified for the 5-marker panel. The results are provided in Table S1.

|  |  |  |  |  | **SN** | **AUC** | **SP90** | **SP95** | **SP98** |
| --- | --- | --- | --- | --- | --- | --- | --- | --- | --- |
| **OPN** | **Resistin** |  |  |  | 94.2 | 0.84 | 0.548 | 0.393 | 0.117 |
| **IL1β** | **OPN** |  |  |  | 94.2 | 0.82 | 0.449 | 0.304 | 0.217 |
| **IFNγ** | **OPN** |  |  |  | 94.9 | 0.77 | 0.379 | 0.314 | 0.247 |
| **OPN** | **MPO** |  |  |  | 94.9 | 0.80 | 0.364 | 0.226 | 0.151 |
| **OPN** | **VCAM** | **Resistin** |  |  | 94.9 | 0.84 | 0.540 | 0.424 | 0.286 |
| **OPN** | **Fibrinogen** | **Resistin** |  |  | 94.9 | 0.83 | 0.500 | 0.365 | 0.237 |
| **OPN** | **MMP7** | **Resistin** |  |  | 94.2 | 0.82 | 0.492 | 0.394 | 0.312 |
| **OPN** | **Resistin** | **APO-B100** |  |  | 94.9 | 0.85 | 0.537 | 0.451 | 0.199 |
| **OPN** | **MMP7** | **VCAM** | **Resistin** |  | 95.7 | 0.82 | 0.533 | 0.436 | 0.326 |
| **IFNγ** | **OPN** | **MMP7** | **MPO** |  | 95.7 | 0.84 | 0.669 | 0.584 | 0.278 |
| **IFNγ** | **OPN** | **MMP7** | **Resistin** |  | 94.9 | 0.82 | 0.586 | 0.463 | 0.343 |
| **OPN** | **MMP7** | **Resistin** | **CRP** |  | 94.9 | 0.83 | 0.474 | 0.393 | 0.250 |
| **IFNγ** | **OPN** | **MMP7** | **Resistin** | **CRP** | 95.7 | 0.83 | 0.639 | 0.501 | 0.274 |
| **IFNγ** | **OPN** | **MMP7** | **Resistin** | **ACRP30** | 94.9 | 0.82 | 0.635 | 0.499 | 0.304 |

**Table S1: Top Ranked Panels Obtained by Cross–validation Testing.** The numerical values indicate the specificity (SP) of the top ranked panels to detect patients without coronary artery disease at sensitivities (90%, 95%, 98%) indicated for the various panels ranging from 2 to 5 markers. The results were determined at sensitivity (SN) of ~95% (94.2%-95.7%) to correctly classify patients with coronary artery disease. AUC: area under ROC curve. (n=239 total, CAD=138, no CAD=101)
